# Pilot validation of blood-based biomarkers during pregnancy and postpartum in women with prior or current depression

**DOI:** 10.1038/s41398-020-01188-4

**Published:** 2021-01-21

**Authors:** E. E. Redei, J. D. Ciolino, S. L. Wert, A. Yang, S. Kim, C. Clark, K. B. Zumpf, K. L. Wisner

**Affiliations:** 1 Department of Psychiatry and Behavioral Sciences, Northwestern University Feinberg School of Medicine, Chicago, IL 60611 USA; 2 The Asher Center, Northwestern University Feinberg School of Medicine, Chicago, IL 60611 USA; 3 Department of Preventive Medicine, Northwestern University Feinberg School of Medicine, Chicago, IL 60611 USA

**Keywords:** Genetics, Biomarkers

## Abstract

Major depressive disorder (MDD) is more common in women than in men, and evidence of gender-related subtypes of depression is emerging. Previously identified blood-based transcriptomic biomarkers distinguished male and female subjects with MDD from those without the disorder. In the present pilot study, we investigated the performance of these biomarkers in pregnant and postpartum women with prior major depressive episodes, some of whom had current symptomatology. The symptom scores of 13 pregnant and 15 postpartum women were identified by the Inventory of Depressive Symptoms (IDS-SR-30) at the time of blood sampling. Blood levels of the 20 transcriptomic biomarkers and that of estrogen receptor 2 (*ESR2*), membrane progesterone receptor alpha and beta (*mPRα*, *mPRβ*) were measured. In pregnant women, transcript levels of *ADCY3*, *ASAH1*, *ATP11C*, *CDR2*, *ESR2*, *FAM46A*, *mPRβ, NAGA*, *RAPH1*, *TLR7*, and *ZNF291/SCAPER* showed significant association with IDS-SR-30 scores, of which *ADCY3*, *FAM46A*, *RAPH1*, and *TLR7* were identified in previous studies for their diagnostic potential for major depression*. ASAH1 and ATP11C* were previously also identified as potential markers of treatment efficacy. In postpartum women, transcript levels of *CAT*, *CD59*, and *RAPH1* demonstrated a trend of association with IDS-SR-30 scores. Transcript levels of *ADCY3*, *ATP11C*, *FAM46A*, *RAPH1*, and *ZNF291/SCAPER* correlated with *ESR2* and *mPRβ* expressions in pregnant women, whereas these associations only existed for *mPRβ* in postpartum women. These results suggest that a blood biomarker panel can identify depression symptomatology in pregnant women and that expression of these biomarker genes are affected by estrogen and/or progesterone binding differently during pregnancy and postpartum.

## Introduction

Depression is the fourth leading contributor to the global burden of disease and the second leading cause of disability in persons 15–44 years of age^1–3^. Depression is twice as common in females as in males, and gender differences in the symptoms and potential etiology of major depressive disorder (MDD) have been described extensively. Although reproductive hormonal differences between men and women have been proposed as explanations for these differences, other factors have also been described. These factors include gender differences in stress-responsiveness and anxiety co-morbidity and the fact that MDD heritability is higher in women (42%) than men (29%)^2^. While the rate of recurrent depression is similar in men and women^4,5^, the prevalence of reproductive hormone-related episodes, such as premenstrual dysphoric disorder, perinatal, and perimenopausal depression, suggest that fluctuating hormone concentrations confer risk specifically for women^6^. Therefore, an increasing genetic contribution to risk for depression may render women particularly vulnerable to develop episodes at times of major biopsychosocial stress, such as during reproductive events.

No valid, practical biological diagnostic tests for MDD have emerged despite decades of neuropsychiatric research. The most commonly used approaches for the diagnosis of MDD are clinician-rated scales and self-report assessments. Although the clinician-rated scales are very costly, they continued to be used in conjunction with self-reported measures^7^. An objective, laboratory-based tool holds promise to increase the diagnostic accuracy of MDD and promote individualized treatment. A diagnostic test would be particularly welcoming in primary care settings, where the majority of patients with MDD are treated^8^. The sensitivity of diagnosis in this setting is 50%, which suggests a large number of missed cases^9^. These and other data suggest that aiding MDD diagnosis in the primary care setting has the potential to significantly and positively affect precision of diagnosis and speed of treatment. Specificity of diagnosis is as essential as its reliability; biological markers correctly identifying MDD patients would greatly contribute to improving both.

A blood test for MDD is particularly appealing to all nonpsychiatric practitioners, who are generally not trained to diagnose or treat MDD. In our prior investigations, we identified blood-based transcriptomic markers that distinguished adolescent and adult subjects with MDD from those without the disorder^10^. A panel of 11 markers differentiated adolescent participants with MDD from those with no disorder. We also evaluated the promising transcript panel in a group of depressed adult subjects before and after a course of treatment with cognitive behavioral therapy (CBT). In that study, blood transcript concentrations of nine markers differed significantly between participants with MDD and no disorder controls at baseline; therefore, they comprise of state-specific markers. Levels of three transcripts remained significantly different between subjects with MDD and controls, even after post-CBT remission, which suggests that they may be trait markers. Further evidence of trait-specificity is that these three transcripts demonstrated high discriminative ability between MDD and control participants, regardless of their contemporaneous clinical status^11^.

Blood levels of our biomarker transcripts differentiated depressed from non-depressed adolescent and adult individuals. However, in our prior studies, we did not explore whether blood levels of these transcripts were associated with symptom severity, but whether they substantiated clinical diagnoses of MDD. Identifying symptom change without changing or defining diagnostic status has potential to enable just-in-time interventions to prevent clinical deterioration. This would be particularly relevant in pregnancy and postpartum, as early and objective identification of depressive symptomatology during pregnancy by a diagnostic tool may speed the provision of treatment and prevent negative effects on the mother and the fetus^12^. Furthermore, the peripartum period increases the risk for recurrence of depressive episodes^13^, possibly due to the effect of hormonal changes during this stressful time.

Although estrogen and progesterone are thought to be causally related to some forms of depression in women^14–16^, the specific mechanism(s) by which it occurs has not been established to date. Of the estrogen receptors, estrogen receptor beta (*ESR2*) has emerged as being expressed in human lymphocytes and playing an extensive role in the nervous system^17,18^. Specifically, it is suggested that estrogen ameliorate depression in women via *ESR2*^19^. Membrane progesterone receptors have gained recognition as targets of progesterone immunosuppressive actions^20–22^. Specifically, both membrane progesterone receptor alpha and beta (Progestin and AdipoQ Receptor 7 and 8, respectively; *PAQR7* = *mPRα* and *PAQR8* = *mPRβ*) are present in human lymphocytes^23^ and *mPRβ* is detected in the nervous system as well^24^. Expression of *mPRα* and *mPRβ* is upregulated following classical progesterone receptor activation^25^, during times of high levels of progesterone. Since both estrogen and progesterone levels change significantly from pregnancy to postpartum, the expression of these receptors may differ between these states and presumably by depression symptoms as well.

In this pilot study, we aimed to explore associations between blood biomarker transcript levels and depressive symptom scores in pregnant and postpartum women with prior and current depressive symptomatology. An additional goal was to determine how blood transcript levels of *ESR2*, *mPRα*, and *mPRβ* are associated with symptom scores and with blood biomarker transcript levels.

## Methods

### Participants

The study included female participants during pregnancy or postpartum from the Assessing Stress, Health, Emotion, and Response Registry in the Asher Center for the Study and Treatment of Depressive Disorders at Northwestern University. Participants consented to optional whole blood collection for RNA isolation as part of the Registry. Participants met criteria for a current or past Major Depressive Episode according to DSM-IV criteria^26^. Samples from enrolled participants meeting these criteria from April 1, 2014 to May 31, 2016 were eligible for analyses.

### Inventory of Depressive Symptoms (IDS-SR-30)

The Inventory of Depressive Symptomatology Self-Report (IDS-SR-30) is a self-rated 30-item questionnaire that assesses the prior seven-day period and covers questions in all domains designated by the DSM-IV to diagnose a major depressive episode. The IDS-SR-30 is used to track severity of depressive symptoms and can also be used for depression screening. Scoring ranges from 0 to 84, with higher scores corresponding to increased depressive symptoms. Scores of 14–25, 26–38, 39–48, and 49–84 represent cut points for mild, moderate, severe, and very severe depressive symptoms, respectively^27^.

Each study participant had a concomitant IDS-SR-30 recorded at the time of sample collection.

### Blood RNA isolation and quantitative RT-PCR

Blood samples were collected into PAXgene blood RNA tubes (Qiagen, Inc.). Samples were stored at −80 °C. Only the randomly assigned code number identified the samples. No information on diagnostic status, identification of serial samples from an individual patient, or time sequence in which the sample was collected were included in the sample labelling. The analytic laboratory is housed in a building separate from the clinical and blood sampling site.

Isolation of blood RNA and quality control were done as described previously^10,11^. Briefly, whole blood total RNA was obtained using the Qiagen PAXgene Blood RNA Kit (Qiagen, Valencia, CA, USA). The yield and quality of extracted RNA was assessed using the NanoDrop™ 1000 spectrophotometer (NanoDrop Technologies, Wilmington, DE). First-strand complementary DNA was synthesized using SuperScript VILO MasterMix (Thermo Fisher Scientific, Carlsbad, CA). Quantitative real-time PCR (qPCR) was carried out using All-in-One™ qPCR Mix (GeneCopoeia, Rockville, MD) and the QuantStudio 7 Flex Real-Time PCR System (Thermo Fisher Scientific, Waltham, MA). A 384-well array was custom-made by GeneCopoeia (Rockville, MD) using the primers described in Supplemental Table 1. *GAPDH* and *ACTB* were used as housekeeping genes. In the analysis of these results, the geometric mean of the fold-changes of *GAPDH* and *ACTB* was calculated and used for normalizing target gene results. Normalized CT values (ΔCT) for each amplified target transcript were calculated.

Transcript levels were measured for these genes: *ADYC3*, *AMFR*, *ASAH1*, *ATP11C*, *CADM1*, *CAT*, *CD59*, *CDR2*, *CMAS*, *DGKA*, *FAM46A*, *KIAA1539/FAM214B*, *MAF*, *MARCKS*, *NAGA*, *PSME1*, *PTP4A3*, *RAPH1*, *TLR7*, *ZNF291/SCAPER*, *ESR2*, *mPRα*, and *mPRβ*.

### Statistical analyses

We focused our analyses on ΔCT values for each sample, and each participant had a corresponding IDS-SR-30 recorded at the time of sample collection. Of note, the dataset included multiple observations on the same participant in several instances, although each sample had a unique IDS-SR-30 score corresponding to the time of sample collection. We computed descriptive statistics for participant-level characteristics—mean (standard deviation) for continuous variables and count (percentage) for categorical variables. Analyses evaluating associations of biomarker transcript levels with IDS-SR-30 scores included samples collected during pregnancy separately from those collected during the postpartum period. Within each subgroup, we evaluated each gene (*ADYC3*, *AMFR*, *ASAH1*, *ATP11C*, *CADM1*, *CAT*, *CD59*, *CDR2*, *CMAS*, *DGKA*, *FAM46A*, *KIAA1539/FAM214B*, *MAF*, *MARCKS*, *NAGA*, *PSME1*, *PTP4A3*, *RAPH1*, *TLR7*, and *ZNF291/SCAPER)* individually for potential associations with symptom measures via a series of individual linear mixed models (LMMs)for IDS-SR-30 with random participant effect and fixed ΔCT effects. The random participant effect allows for distinction between the within versus between participant variance (i.e., it accounts for correlation within an individual). When the model was singular simple linear regression was performed.

Since we sought to determine the association between gene expression profile and IDS-SR-30, we first decomposed the correlation matrices via a principal component analyses (PCA). Then we examined the predictive ability of the principal components (PCs) for IDS-SR-30 via a series of LMMs with random participant effects or linear regression models.

To incorporate the effects of hormonal status on biomarker transcript levels into the analysis, we evaluated associations between biomarker transcript levels and those of *ESR2*, *mPRα*, and *mPRβ* via separate LMMs. We further included these three genes in the correlation matrix and PCA noted above. Statistical analyses were conducted using SAS version 9.4 (The SAS Institute; Cary, NC; 2012) and R (version 3.4.2). All analyses assumed a two-sided 5% level of significance. No adjustments were made for multiple hypothesis testing.

## Results

A total of 39 samples of which 23 samples were from 13 pregnant women and 16 samples from 15 women during the first 6 months of the postpartum period were included in analyses. Table 1 lists the descriptive statistics of the individual participants (*n* = 18). Ten women provided samples both during pregnancy and at postpartum, while three women had samples only during pregnancy, and five women provided samples during the postpartum only. Most pregnant women were sampled more than once at mid- and late pregnancy.

**Table 1:** Sociodemographic characteristics of subjects.

	Overall (*n* = 18)
Age	
Mean (SD)	34.1 (3.25)
BMI	
Mean (SD)	24.3 (3.33)
Race	
White	13 (72.2%)
Black or African American	1 (5.6%)
Other	4 (22.2%)
Education	
Less than College	1 (5.6%)
College	7 (38.9%)
More than College	10 (55.6%)
Employment	
No	7 (38.9%)
Yes	11 (61.1%)
Marital status	
Single, never married	0 (0%)
In a relationship, never married	1 (5.6%)
Married	16 (88.9%)
Separated/Divorced	1 (5.6%)
Sample types	
From pregnant women only	3 (16.7%)
From women at postpartum only	5 (27.8%)
From women during pregnancy and postpartum	10 (55.6%)
Number of samples from pregnant women	
1	4
2	8
3	1
Number of samples from postpartum women	
1	14
2	1

Within the pregnant subgroup, IDS-SR-30 scores were significantly associated with transcript levels of *ADCY3*, *ASAH1*, *ATP11C*, *CDR2*, *ESR2. FAM46A*, *mPRβ, NAGA*, *RAPH1*, *TLR7*, and *ZNF291/SCAPER* (Table 2). Principal component analysis (PCA) was used to decompose the transcript level associations with symptom scores. This allowed us to evaluate the simultaneous expression of these genes for association with IDS-SR-30. Seven principal components explained ~83% of the variation in association between transcript levels and symptom scores. Principal components 1 (34% of variation), 2 (16% of variation), and 4 (7% of variation) were significantly related to IDS-SR-30 in the LMM analyses. Larger PC scores were associated with higher depressive symptom scores. The first and second component, PC1 and PC2, were significantly and positively associated with IDS-SR-30 (*p* = 0.018 and *p* = 0.040; Table 3). The fourth principal component was negatively associated with IDS-SR-30 scores (*p* = 0.037).

**Table 2:** Mixed effect models to evaluate association between IDS-SR30 scores and transcript levels of blood biomarkers in pregnant women.

Delta CT predictor	Estimate	Standard error	*p*-value
***ADCY3***	**6.95**	**2.30**	**0.0132**
*AMFR*	6.62	3.67	0.1031
***ASAH1***	**6.40**	**2.25**	**0.0178**
***ATP11C***	**9.33**	**2.26**	**0.0024**
*CADM1*	3.73	1.88	0.0724
*CAT*	4.24	2.82	0.1621
*CD59*	−1.78	4.48	0.6990
***CDR2***	**12.48**	**4.38**	**0.0178**
*CMAS*	0.61	3.98	0.8812
*DGKA*	8.00	4.70	0.1170
***ESR2***	**12.32**	**5.29**	**0.0414**
***FAM46A***	**8.33**	**3.64**	**0.0451**
*KIAA1539/FAM214B*	1.36	5.96	0.8237
*MAF*	−2.88	3.23	0.3936
*MARCKS*	0.39	4.38	0.9313
*mPRalpha*	1.63	4.10	0.6977
***mPRbeta***	**8.44**	**3.70**	**0.0456**
***NAGA***	**12.35**	**3.99**	**0.0115**
*PSME1*	4.06	5.01	0.4354
*PTP4A3*	2.99	4.92	0.5533
***RAPH1***	**6.87**	**2.31**	**0.0141**
***TLR7***	**6.18**	**2.35**	**0.0258**
***ZNF291/SCAPER***	**7.26**	**1.86**	**0.0035**

Bold terms and values indicate significant association with depression scores.

**Table 3:** Mixed effect model with principal components in sample of pregnant women.

	Reg IDS tot		
Predictors	Estimates	CI	*p*
(Intercept)	22.15	13.89 to 30.41	**<0.001**
Prin 1	1.42	0.55 to 2.30	**0.018**
Prin 2	3.22	0.45 to 5.98	**0.040**
Prin 3	−1.01	−3.45 to 1.43	0.438
Prin 4	−5.83	−10.85 to 0.82	**0.037**
Prin 5	−1.19	−3.37 to 0.98	0.323
Prin 6	−1.71	−4.65 to 1.23	0.292
Observations	23		
Marginal R^2^/conditional R^2^	0.327/0.935		

Bold values indicate significance of principal component.

Transcripts with the highest loadings on PC1were also associated significantly with IDS-SR-30 scores, individually (Table 4). This includes: *ADCY3*, *ASAH1*, *ATP11C*, *CDR2*, *ESR2. FAM46A*, *mPRβ, NAGA*, *RAPH1*, *TLR7*, and *ZNF291/SCAPER*. Only *MAF* and *mPRα* loaded positively on PC2. Thus, simultaneous higher positive loading (higher ΔCT values and therefore lower transcript levels) means that these transcripts and IDS-SR-30 scores have an inverse relationship, concurrently.

**Table 4:** Loadings of transcripts on principal components in pregnant women.

Eigenvectors									
	*PC1*	*PC2*	PC3	PC4	PC5	PC6	PC7	PC8	PC9
***ADCY3***	***0.294609***	0.132693	−0.090982	0.065884	−0.115873	−0.033748	−0.068383	−0.352694	−0.015808
*AMFR*	0.182844	−0.015791	0.315271	**0.283971**	0.198672	−0.234073	0.051532	−0.113376	−0.336138
***ASAH1***	***0.298217***	−0.127616	−0.012742	**−0.285173**	−0.156453	0.000462	0.011648	−0.004317	0.179569
***ATP11C***	***0.330119***	−0.051031	0.072983	0.043465	−0.100023	−0.101293	0.074995	0.114477	−0.209013
*CADM1*	0.196844	0.163432	−0.210282	**0.406636**	0.129485	0.018471	−0.168494	−0.117710	0.304218
*CAT*	0.155144	*−0.273126*	0.309841	0.082877	−0.059024	−0.006353	−0.126168	−0.330794	0.186143
*CD59*	0.122533	*−0.384091*	0.087484	−0.006789	0.069021	0.141872	0.268031	0.200732	−0.267275
***CDR2***	***0.211586***	0.161626	0.340870	0.002337	0.156155	0.045255	0.105101	−0.105640	−0.087187
*CMAS*	0.061593	*−0.312143*	0.258790	0.246783	0.333141	−0.176881	−0.239952	0.160772	0.080353
*DGKA*	0.129550	0.213376	0.158631	**−0.385022**	0.205272	0.350521	0.204440	−0.296923	0.081144
***FAM46A***	***0.239849***	−0.098821	−0.308718	−0.056179	0.259231	−0.108436	0.018303	0.212293	0.269182
*KIAA1539/FAM214B*	0.085405	*−0.331684*	−0.126052	−0.017084	0.447230	0.127239	0.170238	−0.160449	0.222744
*MAF*	0.001101	*0.328408*	−0.032214	0.222571	0.378790	0.205172	0.317436	0.298592	0.065712
*MARCKS*	0.161913	*−0.377031*	−0.135346	−0.087677	−0.142381	0.156507	0.263153	0.042090	−0.096970
***NAGA***	***0.231321***	0.108171	0.132499	0.007888	−0.227001	−0.304011	0.221883	0.446774	0.248449
*PSME1*	0.151892	0.140626	0.140247	**0.315208**	−0.262034	0.494897	0.197269	0.112543	0.113270
*PTP4A3*	0.158059	0.031393	−0.372175	**0.294017**	−0.189874	−0.273052	0.285048	−0.261245	0.010067
***RAPH1***	***0.290029***	0.088920	−0.086147	−0.197964	−0.026889	−0.155580	0.119822	0.022125	−0.272393
***TLR7***	***0.249289***	0.006171	0.172625	−0.146444	−0.152377	0.145925	−0.400464	0.258639	0.267893
***ZNF291/SCAPER***	***0.316485***	0.120942	0.121851	0.016653	−0.013266	0.010604	−0.080651	−0.147327	0.042069
***ESR2***	***0.228543***	0.053197	−0.288676	**−0.277135**	0.228711	−0.059148	−0.155323	0.025412	−0.055382
*mPRα*	−0.079699	*0.282425*	0.244249	−0.255568	0.165357	−0.412689	0.189740	−0.012688	0.136649
***mPRβ***	***0.202668***	0.194737	−0.171126	0.028660	0.138277	0.176741	−0.387622	0.169489	−0.460027

Bold plus italics are for high loading transcripts on PC1, italics are for high loading transcripts on PC2 that are unique to PC2, and bold ones for PC4.

In contrast, PC2 contains several transcripts with negative loadings—specifically *CAT*, *CD59*, *CMAS*, *KIAA1539/FAM214B*, and *MARCKS—*which overlap neither with those loading heavily on PC1, nor with those that were individually associated with IDS-SR-30. This suggests that simultaneous increased expression of these genes is associated with higher IDS-SR-30. Interestingly, PC4 was significantly (*p* = 0.037), but negatively associated with IDS-SR-30, and it had the highest positive loadings for transcripts *AMFR*, *CADM1*, *PSME1*, and *PTP4A3*, which did not overlap with the heavy loading of either PC1 or PC2. In the negative relationship between PC4 and IDS-SR-30, positive contribution to PC4 associates with lower IDS-SR-30 scores. Thus, simultaneously high ΔCT values for these genes (i.e., lower expression) is associated with lower IDS-SR-30 score.

Examining the correlation between the biomarker transcript levels and that of *ESR2*, *mPRα* and *mPRβ*, resulted in a notable pattern (Table 5). Only three transcripts (*CDR2*, *NAGA*, and *TLR7)* among those that showed significant association with IDS-SR30 scores were not associated with *ESR2* and concurrently with *mPRβ* expression. All of the significant relationships noted between biomarker expressions and *ESR2* and *mPRβ* transcript levels were positive. In contrast, *mPRα* showed negative association with *MARCKS* transcript levels. *FAM46A*, *TLR7*, and *PSME1* were the only transcripts that were not significantly linearly associated with any of these hormone receptors in this dataset.

**Table 5:** Mixed effects model to evaluate association between biomarker transcript levels and expression of estrogen receptor 2 (*ESR2*), membrane progesterone receptor alpha (*mPRα*), and beta (*mPRβ*) in pregnant women.

	*ESR2*	*mPRα*	*mPRβ*
*ADCY3*	1.12**	*−0.13*	0.81**
*AMFR*	0.12	0.02	0.15
***ASAH1***	1.05**	***−0.25***	***0.5***
*ATP11C*	0.76*	*−0.19*	0.5*
*CADM1*	1.71**	*−0.34*	1.33**
*CAT*	−0.04	−0.24	−0.16
*CD59*	0.38	−0.29	0.1
*CDR2*	0.12	0.14	0.27
*CMAS*	0.1	−0.05	−0.02
*DGKA*	0.35	0.19	0.27
*FAM46A*	1.13***	*−0.27*	0.69**
***KIAA1539/FAM214B***	0.62**	***−0.23***	***0.1***
*MAF*	0.06	0.28	0.4
*MARCKS*	0.59	−0.59**	0.19
*NAGA*	0.35	0.08	0.21
*PSME1*	0.02	−0.1	0.19
*PTP4A3*	0.47	−0.24	0.27
*RAPH1*	1.37***	*−0.01*	0.82**
*TLR7*	0.69	−0.14	0.53
*ZNF291/SCAPER*	0.97**	*0*	0.75**

Bold plus italics significant correlations with *ESR2* only, italics significant correlations with both *ESR2* and *mPRβ*.

*p < 0.05.

**p < 0.01.

***p < 0.001.

The association between IDS-SR-30 scores and biomarker transcript levels were different in postpartum samples from those in pregnant women. Although none of the associations between biomarker transcript levels and IDS-SR-30 were statistically significant, *CAT*, *CD59*, and *RAPH1* were marginally associated at a more relaxed level of significance (*p* < 0.1; Supplemental Table 2). The PCA within the postpartum samples revealed five principal components, which explained ~82% of the variation, but none of these components associated with IDS-SR scores significantly.

In contrast to the many significant associations noted between *ESR2* and *mPRβ* and biomarker transcript levels in pregnant women, only *CADM1* and *FAM46A* transcript levels were significantly associated with *ESR2* and *mPRβ* simultaneously in postpartum samples (Table 6). However, *ADCY3*, *ASAH1*, *ATP11C*, *CDR2*, *CMAS*, *DGKA*, *MARCKS*, *NAGA*, *RAPH1*, and *ZNF291/SCAPER*, were all significantly associated with *mPRβ* alone, of which correlations with *ASAH1*, *CDR2*, *CMAS*, *DGKA*, *MARCKS*, and *NAGA* were unique to the postpartum samples. All relationships noted between biomarker expression and *mPRβ* transcript levels were positive with the exception of *MARCKS*. Expression of *PTP4A3* was positively associated with *mPR*α. In this dataset, the following transcripts were not significantly associated with any of these hormone receptors: *AMFR*, *CAT*, *CD59*, *KIAA1539/FAM214B*, *MAF*, *PSME*, and *TLR7*.

**Table 6:** Linear regression models to evaluate association between biomarker transcript levels and expression of estrogen receptor 2 (*ESR2*), membrane progesterone receptor alpha (*mPRα*), and beta (*mPβ*) in postpartum samples.

Transcripts	*ESR2*	*mPRα*	*mPRβ*
*ADCY3*	0.22	−0.04	0.33**
*AMFR*	0.1	0.06	0.08
*ASAH1*	0.25	−0.26	0.43**
*ATP11C*	0.33	−0.01	0.4**
*CADM1*	0.72*	0.11	0.75**
*CAT*	0.13	−0.2	0.16
*CD59*	−0.21	−0.32	0.14
*CDR2*	0.22	−0.02	0.29**
*CMAS*	0.27	−0.07	0.31*
*DGKA*	0.24	−0.22	0.27*
*FAM46A*	0.42**	0.25	0.35**
*KIAA1539/FAM214B*	0.2	0.32	−0.12
*MAF*	0.05	−0.37	0.2
*MARCKS*	−0.11	0.1	−0.29*
*NAGA*	0.23	0.11	0.26*
*PSME1*	0.18	0.14	0.16
*PTP4A3*	0.23	0.42*	0.12
*RAPH1*	0.61	0.2	0.63**
*TLR7*	0.39	0.55	0.41
*ZNF291.SCAPER*	0.48	0.13	0.68**

Estimates and significance are obtained from separate linear regression models.

*p < 0.05.

**p < 0.01.

To determine the association between progesterone receptor levels and pregnancy status either ad hoc LMM with random subject effect (*mPRα*) or linear regression analysis due to singularity (*ESR2* and *mPRβ*) was performed. Based on a two-sided 5% significance level, there was not enough evidence to suggest that *ESR2* transcript levels are different at pregnancy and postpartum visits. In contrast, *mPR*α and *mPRβ* levels were significantly different between pregnancy and postpartum visits, specifically lower at postpartum (*p* = 0.041 and *p* = 0.027, respectively).

## Discussion

This pilot study involved a clinical sample of pregnant and postpartum women with prior episodes of major depression and differing severity of current symptomatology. We found that depression symptom severities were associated with the expression of specific blood-based transcriptomic markers in pregnant women. In contrast, only a trend of association between IDS-SR symptom scores and different biomarkers could be observed in postpartum samples. This difference between the relatedness of symptom severity with biomarker transcript levels in the blood was surprisingly echoed by the difference in how the expression of these biomarkers correlated with estrogen and membrane progesterone receptor transcript levels during pregnancy and postpartum.

We did not expect significant overlap between the results of the present and the previous studies using the same blood markers^10,11^. This expectation was a result of the current study design including samples from pregnant and postpartum women with obvious differing hormonal environments, a continuous pattern of symptom severity rather than a discrete depressed/not-depressed clinical diagnosis, and excluding a comparison group without prior MDD. However, the generalizability of some of the biomarkers as state markers was still anticipated. In our previous study of adult primary care subjects, blood transcript levels of *ADCY3*, *DGKA*, *FAM46A*, *CADM1*, *FAM214B*, *MARCKS*, *PSME1*, *RAPH1*, and *TLR7* differed significantly between participants with MDD compared to matching no disorder controls^11^. Transcript levels of all these genes were higher (the ΔCT lower) in the blood of the MDD group. In the current study, *ADCY3*, *ASAH1*, *ATP11C*, *CDR2*, *FAM46A*, *NAGA*, *RAPH1*, *TLR7*, and *ZNF291/SCAPER* correlated significantly with IDS-SR30 scores in pregnant women. Some of these transcripts, namely *ADCY3*, *FAM46A*, *RAPH1*, *TLR7*, therefore, overlap in their state marker characteristics with those we observed previously. This overlap occurred despite the small number of pregnant women in the study. However, findings for the postpartum samples showed only a trend of association between the expressions of *CAT*, *CD59*, and *RAPH1* blood markers and depression scores. To argue for the small sample size as a reason for this finding in postpartum women is at odds with the very significant and weighty results in the pregnant samples. However, individual sensitivity to decreased estrogen and progesterone levels after birth, and the effect of this hormone milieu on blood marker levels of individual postpartum women, is a potential mechanism for these results.

The present data gives an insight into the long-predicted association between peripheral estrogen (primarily estradiol, E2) and progesterone (P4) levels and women’s increased vulnerability to hormonal change-associated affective disorders. In pregnant women, with their known dramatic increase in E2 and P4 levels, blood transcript levels of both *ESR*2 and *mPRβ* were significantly correlated with IDS-SR-30 scores. Results of the principal component analysis suggested an inverse relationship between expression of these two receptors in the blood and depressive symptoms. Thus, lower levels of these receptor transcripts were associated with higher depression scores. Since expression of these receptors are positively regulated by their ligands^28,29^, their lower levels during the depressed state would imply decreased levels of E2 and P4 in depressed women during pregnancy or decreased sensitivity of the receptors to their ligands.

In postpartum women, there was no association between symptom scores and hormone receptor levels in this dataset. This is in agreement with the failure to demonstrate an association between ovarian hormones levels and depressive symptoms during the postpartum period^30^. In contrast, studies in which postpartum depression was treated with estradiol have successfully reduced depressive symptoms^31^. Even more, the neurosteroid metabolite of progesterone—allopregnanolone—regulates neuronal function and may mediate affective dysregulation that occurs concomitant with changes in reproductive endocrine function^32^. Allopregnanolone is the first FDA-indicated drug for postpartum depression^33^. Thus, the question of this seeming paradox can be examined in the light of the dramatic decrease in peripheral E2 and P4 levels postpartum, and our current findings that correlation of biomarker expression shifted from that of concomitant *ESR2* and *mPRβ* in pregnancy to *mPRβ* alone during postpartum.

Estrogen and progesterone regulate the expression of the *mPR* genes^34,35^; E2 can regulate the expression of *mPRβ* both in the periphery and in the brain^36^, while P4 can do that only in the brain^37^. Progesterone synthesis in the brain is induced by E2 from gonadal origin^38,39^, and thus it is feasible that gonadal E2 is necessary, but not sufficient, in the regulation of mood for P4 effects on depressive symptoms via *mPRβ*. When E2 and P4 levels are high, such as during pregnancy, expressions of biomarkers correlated positively with *mPRβ* in parallel with *ESR2*. When levels of both these hormones are low, such as postpartum, *mPRβ* levels are decreased in the blood, and expressions of most of the biomarkers correlated with *mPRβ* and not *ESR2* in postpartum samples. Decreased E2 of gonadal origin would also decrease the synthesis of P4 in the brain. This is significant, since through its neuroactive metabolite, allopregnanolone, P4 modulates the responsiveness to GABAa receptors^40^, which may contribute directly to the etiology of MDD^41,42^. Furthermore, a recent study shows a significant association between low levels of allopregnanolone and the development of postpartum depression in women with a history of mood disorders^43^. Should decreased *mPRβ* levels in the periphery and their correlation with the blood markers be an indication of the role of lower brain P4 levels in postpartum will need to be explored in the future.

Limitations of this pilot study include the small sample size, and the lack of matching subjects with others having no prior or present history of MDD. Another limitation includes lack of data from patients with other psychiatric disorders, and no change in transcript levels were assignable to administration of medication. However, these data suggest that this panel of blood-based biomarkers are associated with concurrent depression symptoms in pregnant women. The study also suggests a relationship between transcript levels of a panel of depression-associated biomarkers and estrogen-dependent and independent expression of membrane progesterone receptors, which relationship differs between pregnant and postpartum samples. This latter finding offers a novel understanding of the specific vulnerability of women to depression at times of major hormonal changes.

## Supplementary information

Supplemental Table S1

Supplemental Table S2
