# Supplementary material for: Pilot validation of blood-based biomarkers during pregnancy and postpartum in women with prior or current depression
Source: Transl Psychiatry. 2021 Jan 21;11:68. doi: 10.1038/s41398-020-01188-4 (PMC7820442; doi:10.1038/s41398-020-01188-4)
Supplement: Supplementary file 1 — Supplemental Table S1 [file 41398_2020_1188_MOESM1_ESM.docx]

**Supplemental Table S1. Mean measures**

|  | Pregnant | Postpartum |
| --- | --- | --- |
| IDS |  |  |
| Mean (SD) | 23.3 (18.1) | 19.8 (15.7) |
| Median [Min, Max] | 18.0 [6.00, 72.0] | 15.0 [5.00, 65.0] |
| *ADCY3* |  |  |
| Mean (SD) | 8.74 (0.587) | 8.43 (0.402) |
| Median [Min, Max] | 8.92 [7.80, 9.60] | 8.37 [7.92, 9.21] |
| *AMFR* |  |  |
| Mean (SD) | 5.62 (0.425) | 5.75 (0.357) |
| Median [Min, Max] | 5.52 [4.96, 6.51] | 5.80 [5.19, 6.50] |
| *ASAH1* |  |  |
| Mean (SD) | 3.72 (0.606) | 4.20 (0.520) |
| Median [Min, Max] | 3.69 [2.37, 4.93] | 4.14 [2.96, 5.36] |
| *ATP11C* |  |  |
| Mean (SD) | 6.44 (0.457) | 6.38 (0.457) |
| Median [Min, Max] | 6.37 [5.67, 7.82] | 6.33 [5.63, 7.08] |
| *CADM1* |  |  |
| Mean (SD) | 11.0 (0.985) | 10.7 (0.871) |
| Median [Min, Max] | 11.2 [8.53, 12.6] | 10.9 [8.70, 12.5] |
| *CAT* |  |  |
| Mean (SD) | 4.53 (0.662) | 4.85 (0.401) |
| Median [Min, Max] | 4.60 [2.97, 5.72] | 4.91 [4.12, 5.36] |
| *CD59* |  |  |
| Mean (SD) | 5.53 (0.591) | 6.04 (0.490) |
| Median [Min, Max] | 5.49 [4.11, 6.84] | 5.98 [5.21, 7.05] |
| *CDR2* |  |  |
| Mean (SD) | 6.91 (0.324) | 6.39 (0.362) |
| Median [Min, Max] | 6.92 [6.38, 7.46] | 6.31 [5.89, 7.26] |
| *CMAS* |  |  |
| Mean (SD) | 7.85 (0.611) | 8.60 (0.424) |
| Median [Min, Max] | 7.86 [6.57, 9.14] | 8.54 [7.96, 9.38] |
| *DGKA* |  |  |
| Mean (SD) | 4.79 (0.389) | 4.27 (0.374) |
| Median [Min, Max] | 4.81 [3.98, 5.49] | 4.24 [3.78, 5.02] |
| *FAM46A* |  |  |
| Mean (SD) | 7.28 (0.438) | 7.33 (0.437) |
| Median [Min, Max] | 7.37 [6.11, 8.27] | 7.45 [6.38, 7.92] |
| *KIAA1539*.*FAM214B* |  |  |
| Mean (SD) | 5.10 (0.417) | 5.46 (0.518) |
| Median [Min, Max] | 5.05 [4.19, 6.09] | 5.53 [4.48, 6.39] |
| *MAF* |  |  |
| Mean (SD) | 10.8 (0.553) | 10.1 (0.546) |
| Median [Min, Max] | 10.8 [9.58, 11.8] | 10.0 [9.21, 11.2] |
| *MARCKS* |  |  |
| Mean (SD) | 7.87 (0.618) | 8.47 (0.419) |
| Median [Min, Max] | 7.83 [6.64, 8.87] | 8.62 [7.42, 9.02] |
| *NAGA* |  |  |
| Mean (SD) | 7.04 (0.377) | 6.90 (0.411) |
| Median [Min, Max] | 7.04 [6.39, 8.01] | 6.94 [6.09, 7.83] |
| *PSME1* |  |  |
| Mean (SD) | 4.21 (0.364) | 4.17 (0.369) |
| Median [Min, Max] | 4.29 [3.41, 4.72] | 4.20 [3.57, 4.90] |
| *PTP4A3* |  |  |
| Mean (SD) | 8.53 (0.474) | 8.23 (0.427) |
| Median [Min, Max] | 8.57 [7.58, 9.42] | 8.21 [7.21, 8.97] |
| *RAPH1* |  |  |
| Mean (SD) | 9.61 (0.644) | 9.11 (0.840) |
| Median [Min, Max] | 9.77 [8.20, 11.1] | 9.18 [7.33, 10.7] |
| *TLR7* |  |  |
| Mean (SD) | 7.52 (0.580) | 7.62 (0.772) |
| Median [Min, Max] | 7.62 [6.34, 8.69] | 7.65 [6.10, 9.20] |
| *ZNF291*.*SCAPER* |  |  |
| Mean (SD) | 7.81 (0.563) | 7.77 (0.785) |
| Median [Min, Max] | 7.66 [7.12, 8.93] | 7.65 [6.76, 9.96] |
| *ESR2*_ |  |  |
| Mean (SD) | 8.57 (0.317) | 8.47 (0.649) |
| Median [Min, Max] | 8.57 [7.93, 9.33] | 8.55 [6.84, 9.91] |
| *mPRalpha*_ |  |  |
| Mean (SD) | 8.39 (0.562) | 7.81 (0.612) |
| Median [Min, Max] | 8.31 [7.64, 9.87] | 7.97 [6.80, 8.99] |
| *mPRbeta*_ |  |  |
| Mean (SD) | 7.70 (0.411) | 7.48 (0.833) |
| Median [Min, Max] | 7.58 [7.06, 8.67] | 7.19 [6.16, 9.10] |
