# Supplementary material for: Pilot validation of blood-based biomarkers during pregnancy and postpartum in women with prior or current depression
Source: Transl Psychiatry. 2021 Jan 21;11:68. doi: 10.1038/s41398-020-01188-4 (PMC7820442; doi:10.1038/s41398-020-01188-4)
Supplement: Supplementary file 2 — Supplemental Table S2 [file 41398_2020_1188_MOESM2_ESM.docx]

| **Supplemental Table 2**. Linear regression to determine association between IDS-SR30 scores and biomarker transcript levels in postpartum women | | | |
| --- | --- | --- | --- |
| ***DeltaCT*** | **Estimate** | **Standard Error** | **p-value** |
| *ADCY3* | 7.27 | 10.94 | 0.5179 |
| *AMFR* | -0.08 | 11.82 | 0.9945 |
| *ASAH1* | 0.50 | 8.24 | 0.9525 |
| *ATP11C* | 2.92 | 9.44 | 0.7623 |
| *CADM1* | -2.29 | 4.80 | 0.6411 |
| *CAT* | -19.48 | 10.74 | 0.0928 |
| *CD59* | -14.68 | 7.57 | 0.0746 |
| *CDR2* | 7.92 | 11.63 | 0.5076 |
| *CMAS* | -11.70 | 10.30 | 0.2766 |
| *DGKA* | 5.85 | 11.91 | 0.6318 |
| *FAM46A* | 1.56 | 9.67 | 0.8740 |
| *KIAA1539/FAM214B* | -1.54 | 8.52 | 0.8596 |
| *MAF* | 0.07 | 8.49 | 0.9932 |
| *MARCKS* | 9.60 | 9.78 | 0.3440 |
| *NAGA* | 14.42 | 9.50 | 0.1528 |
| *PSME1* | 12.50 | 10.89 | 0.2715 |
| *PTP4A3* | 1.09 | 9.90 | 0.9141 |
| *RAPH1* | 8.00 | 4.51 | 0.0993 |
| *TLR7* | 5.28 | 5.33 | 0.3398 |
| *ZNF291/SCAPER* | -1.66 | 5.41 | 0.7635 |
| *ESR2* | 1.70 | 6.67 | 0.8026 |
| *mPRralpha* | -1.29 | 7.05 | 0.8581 |
| *mPRbeta* | -1.91 | 5.07 | 0.7120 |
